# INTERVENTIONS TO PREVENT THE FRAILTY IN OLDER WOMEN WITH FRAILTY: A SYSTEMATIC REVIEW AND META-ANALYSIS

**DOI:** 10.1093/geroni/igac059.2120

**Published:** 2022-12-20

**Authors:** Ha Na Jeong, Sun Ju Chang, Joo Ri Kim, Gi Won Choi

**Affiliations:** Seoul National University, Seoul, Seoul-t'ukpyolsi, Republic of Korea; Seoul National University, Seoul, Seoul-t'ukpyolsi, Republic of Korea; Seoul National University, Seoul, Seoul-t'ukpyolsi, Republic of Korea; Seoul National University, Seoul, Seoul-t'ukpyolsi, Republic of Korea

## Abstract

**Background:**

Frailty is a health challenge related to adverse health outcomes in older adults. Older women are more likely to be frail than older men. However, few studies have reviewed the effectiveness of interventions for older women with frailty is scant. Objective: This systematic review aimed to explore the properties of interventions and to investigate their effectiveness in preventing the progression of frailty in older women with pre-frailty or frailty.

**Methods:**

Narrative synthesis was conducted to identify the contents, outcome variables, and findings of the interventions. Then, a meta-analysis was performed to evaluate the effectiveness of exercise interventions on grip strength, sit-and-reach, sit-to-stand, and timed up and go tests.

**Results:**

Twenty-six studies were selected, including 14 randomized controlled trials and 12 quasi-experimental studies. These studies implemented exercise (96.2%), nutrition (15.4%), hormone replacement (7.7%), toileting strategies (3.8%), and laughter interventions (3.8%). The selected studies assessed physical, psychological (11.5%), and cognitive health (11.5%), as well as quality of life (19.2%). The meta-analysis found significant effects of aerobic and resistance exercise interventions on the sit-to-stand (SMD = 1.30, 95% CI [0.70, 1.90], p < 0.001) and timed up and go scores (SMD = -0.56, 95% CI [-0.93, -0.19], p = 0.003).

**Conclusion:**

Exercise interventions are essential to improve physical health, in particular mobility, in older women with pre-frailty or frailty. Future studies should consider theoretical frameworks and evaluate psychological and cognitive health as well as quality of life to develop and provide effective interventions to prevent the progression of frailty in older women.

